# Authentication of Primary Murine Cell Lines by a Microfluidics-Based Lab-On-Chip System

**DOI:** 10.3390/biomedicines8120590

**Published:** 2020-12-09

**Authors:** Yingfen Hong, Nikita Singh, Stefanos Bamopoulos, Enio Gjerga, Laura K. Schmalbrock, Karl Balabanian, Markus Schick, Ulrich Keller, Matthias Wirth

**Affiliations:** 1 Department of Hematology, Oncology and Tumor Immunology, Campus Benjamin Franklin, Charité–Universitätsmedizin Berlin, 12203 Berlin, Germany; yingfen.hong@tum.de (Y.H.); nikita.nikita@charite.de (N.S.); stefanos.bamopoulos@charite.de (S.B.); enio.gjerga@charite.de (E.G.); laura.schmalbrock@charite.de (L.K.S.); markus.schick@charite.de (M.S.); 2 TUM School of Medicine, Technical University Munich, 81675 Munich, Germany; 3 Institut de Recherche Saint Louis, Université de Paris, EMiLy, Inserm U1160, F-75010 Paris, France; karl.balabanian@inserm.fr; 4 German Cancer Research Center (DKFZ), 69120 Heidelberg, Germany; 5 German Cancer Consortium (DKTK), 69120 Heidelberg, Germany; 6 Max-Delbrück-Center for Molecular Medicine, 13092 Berlin, Germany

**Keywords:** authentication, FLA, STR-PCR, bioanalyzer, mouse cell lines, HoxB8

## Abstract

The reliable authentication of cell lines is a prerequisite for the reproducibility and replicability of experiments. A common method of cell line authentication is the fragment length analysis (FLA) of short-tandem repeats (STR) by capillary electrophoresis. However, this technique is not always accessible and is often costly. Using a microfluidic electrophoresis system, we analyzed the quality and integrity of different murine cell lines by STR profiling. As a proof of concept, we isolated and immortalized hematopoietic progenitor cells (HPC) of various genotypes through retroviral transduction of the fusion of the estrogen receptor hormone-binding domain with the coding sequence of HoxB8. Cell lines were maintained in the HPC state with Flt3 ligand (FL) and estrogen treatment and could be characterized upon differentiation. In a validation cohort, we applied this technique on primary mutant Kras-driven pancreatic cancer cell lines, which again allowed for clear discrimination. In summary, our study provides evidence that FLA of STR-amplicons by microfluidic electrophoresis allows for stringent quality control and the tracking of cross-contaminations in both genetically stable HPC lines and cancer cell lines, making it a simple and cost-efficient alternative to traditional capillary electrophoresis.

## 1. Introduction

Advances in 2- and 3D cell culture technology made cell lines frequently used tools for various biomedical investigations. All blood cells are derived from hematopoietic progenitor cells (HPCs), and, depending on activating cytokines, HPCs can take the path of both myeloid and lymphoid differentiation [1]. Transcription factors of the homeobox (*Hox*) gene family orchestrate differentiation of all hematopoietic lineages and are expressed during HPC self-renewal. Enforced expression of Hox proteins, including HoxB8 can block hematopoietic differentiation [2]. The fusion of HoxB8 and the estrogen receptor (ER-HoxB8) in combination with Flt3 ligand (FL) can be used to conditionally immortalize HPCs (HoxB8-FL HPC), which under these conditions can be cultured with an unlimited proliferative capacity [3]. Due to this characteristic, it is possible to generate large cell numbers for gain- and loss-of-function studies and perform global large-scale analyses by sampling the bone marrow of a single mouse [3]. Through specific growth factors and a co-culture with stromal cells, HoxB8-FL HPCs can differentiate into various hematopoietic lineages, such as B- and T-lymphocytes [3], dendritic cells [4], osteoclasts [5] and granulocytes [6] both in vitro and in vivo. This permits the study of various aspects of hematopoiesis, the immune system and hematopoietic malignancies on a large scale even in rare cell types. The method thus has enormous potential, especially with regard to the 3R principles, i.e., the reduction, replacing and refinement of in vivo models [7].

Non-reproducibility and non-replicability are often the result of a lack of quality controls that guarantee the authenticity of the cell lines used [8]. It is highly risky to conduct experiments with unauthenticated cell lines and to publish the results with misidentified or cross-contaminated cell lines, as found results are not reproducible. Therefore, cell line authentication is inevitable for integrity research [9]. For human cell lines, the standard method for short-tandem repeat (STR) profiling is capillary gel electrophoresis [10]. However, the authentication of “in house”-generated murine cell lines is not yet widely applied. Since murine cell lines may be affected by cross-contamination, it is urgently necessary to build in appropriate quality controls also for murine cell lines generated “in house”. The gold standard STR profiling for human cells can be applied to murine cell lines as shown by Almeida et al. [11]. A limitation of cell line authentication by means of fragment length analysis (FLA) from appropriate STR markers is the inability of rapid on-site analysis. STR profiling by capillary gel electrophoresis is usually outsourced to a third party, often for-profit institutions, which makes frequent authentication of “in house”-generated lines expensive. Therefore, authentication may not be performed on a regular basis. For FLA of defined repeat sequences, it has been shown that a microfluidics-based lab-on-chip system to perform genotyping by STR-polymerase chain reaction (PCR) can be used as an alternative to capillary electrophoresis for forensic samples [12] and human cell lines [11].

First, we generated HPC cell lines using the HoxB8 technology from three different genotypes with identical strain backgrounds and characterized them by flow cytometry and RNAseq to create a basis for the subsequent authentication by STR profiling. Since STR profiling by using the standard method of capillary gel electrophoresis is very time- and cost-intensive, we have established an analytical method using a microfluidic-based lab-on-chip system (Agilent Bioanalyzer 2100), which allows for the on-site authentication of primary murine cell lines. This represents one important cornerstone to generate reproducible and replicable data from “in house”-generated primary murine cell lines and the basis to build quality-controlled living biobanks.

## 2. Materials and Methods

### 2.1. Animal Experiments

All mice were bred and housed at the central breeding facility of Charité under specific pathogen-free conditions. The mice included *wild-type, Iμ-HA-Bcl6* [13] and *Cxcr4^WHIM^* [14]. *Iμ-HA-Bcl6* mice were kindly provided by Prof. Laura Pasqualucci (Columbia University, New York, NY, USA) and *Cxcr4^WHIM^* mice were obtained from Prof. Karl Balabanian (Université Paris-Sud, Paris, France). Three mice for each genotype were sacrificed at the age of 12–14 weeks and bone marrow cells were isolated from the femurs and tibias by a recently described method [15]. Mice were anesthetized by isoflurane administration and subsequently euthanized by cervical dislocation. The light/dark cycle was adjusted to 14 h lights on and 10 h lights off with the start of the light and dark period set at 6.00 am and 8.00 pm, respectively. The Animal Core Facility has regularly tested the health status of mice. All the experiments were conducted in accordance with the animal welfare regulations reviewed and approved by the institutional review board and the Landesamt für Gesundheit und Soziales Berlin (T 0313-18; 04/2019).

### 2.2. Reagents, Plasmids and Media

The following antibodies and staining reagents were used in this study: PE-Cyanine7 anti-mouse B220 (RA3–6B2) (# 25-0452-82), APC-Cy7 anti-mouse MHC class II (M5/114.15.2) (# 47-5321-82), eFluor450 anti-mouse GR1 (Ly6G (RB6–8C5) (# 48-5931-82), PE-Cy5 anti-mouse CD11c (N418) (# 45-0114-82), FITC anti-mouse Sca-1 (D7) (# 11-5981-85), FITC anti-mouse CD11b (M1/70) (# 11-0112-85), APC anti-mouse c–Kit (2B8) (# 17-1171-82),eFluor450-CD19 (1D3) (# 48-0193-82), were purchased from eBioscience™ (Santa Clara, CA, USA). ELISA kits for mouse FLT3 Ligand (# EMFLT3L) were from Thermo scientific (Darmstadt, Germany). Recombinant mouse growth factors (SCF, FLT3L, IL-3, IL-6, IL-7) were from R & D Systems (Minneapolis, MN, USA). Polybrene (# TR-1003-G) was purchased from Millipore (Burlington, NJ, USA). Lipofectamine 2000 (# 11668019) was purchased from Invitrogen (Waltham, MA, USA). G418 (# 4727878001) was purchased from Roche (Basel, Switzerland). ACK (Ammonium-Chloride-Potassium) lysing buffer (A10492-01) was purchased from Gibco^TM^ (Darmstadt, Germany). *MSCV-neo–HA-ERHBD–HOXB8* plasmid was kindly provided by Hans Häcker (St. Jude Children’s Research Hospital, Memphis, TN, USA). The media (Table 1) were prepared according to the publication by Redecke et al. [3].

### 2.3. Surface Marker Detection by FACS

Cells were washed three times with PBS and resuspended in HF2 Buffer. For dendritic cell (DC) and macrophage evaluation, cells were stained with eFluor450 anti-mouse GR1 (Ly6G (RB6–8C5) (1:400), FITC anti-mouse CD11b (M1/70) (1:20), APC anti-mouse c–Kit (2B8) (1:200). For B-cell evaluation, cells were stained with PE-Cyanine7 anti-mouse B220 (RA3–6B2) (1:50), CD19 (1D3) (1:200). After incubating for 45 min at 4 °C in the dark, cells were washed and stained with Propidiumiodide (1:10,000 of a 1 mg/mL stock solution) for the exclusion of dead cells. Data were analyzed using the FlowJo software (V10.6.1).

### 2.4. Virus Production

The *MSCVneo–HA-ERHBD–HOXB8* vector was transfected into ecotropic retroviral packaging cells (PhoenixTM-Eco, ThermoFisher, Waltham, MA, USA) using Lipofectamine 2000 (Invitrogen) according to the manufactures’ protocol. A total of 12 h after transfection, the supernatant (SN) was replaced by fresh BBMM. Virus–containing supernatants are collected 36, 48, and 72 h after transfection and filtered using a 0.45 μM filter and stored at 4 °C until further usage for transduction of immortalized ER-HoxB8 cells within 2 weeks.

### 2.5. Cell Lines

The Phoenix™-Eco cell line (CRL-3214™) and MM.1S cell line (CRL-2974™) were purchased from American Type Culture Collection (ATCC) (Manassas, VA, USA). The OP9 cell line, NIH3T3 cell line, and Flt3-Ligand-producing B16 melanoma cell line were kindly provided by Prof. Dr. Marc Schmidt-Supprian (Technische Universität München, Munich, Germany).

Phoenix™-Eco cells were maintained in high-glucose DMEM (Thermo Fisher, # 11965084) supplemented with 10% FBS, 1% L-Glutamin and 1% Antibiotic Antimycotic (Anti-Anti, # 15240-062, Gibco). To passage, cells were washed in 2 mL PBS, then incubated with 0.2 mL of 1×Trypsin (diluted in PBS) for two minutes at 37 °C. Cells were collected and centrifuged at 400 relative centrifugal force (RCF) for five miHEPESnutes to pellet followed by inactivating trypsin with 9 mL of high-glucose DMEM. The supernatant was aspirated and the pellet was resuspended in high-glucose DMEM/1% L-Glutamin/10% FCS/1% Anti-Anti. Cells were seeded at a low density in 10 cm dishes and passaged every four days.

The Flt3 Ligand (Flt3L)-producing B16 melanoma cell line was maintained in high-glucose DMEM supplemented with 10% FBS and 1% Anti-Anti. Cells were seeded in T75 flasks and passaged every 3–4 days. To passage, cells were washed in 10 mL of PBS twice, then incubated with 1 mL of 1×Trypsin for 5 min at 37 °C. Cells were collected and centrifuged at 400 RCF for five minutes to pellet followed by inactivating trypsin with 15 mL of high-glucose DMEM. The supernatant was collected, filtered by 0.45 µM filters for Flt3L measurement and stored at −20 °C, and the pellet was resuspended in high-glucose DMEM/10% FBS/1% Anti-Anti. Cells were replated at a low density in 10 mL of high-glucose DMEM for culturing. The OP9 cell line was cultured in Alpha Minimum Essential Medium (# 12561056, Gibco)/20% FBS/1% Anti-Anti (15240-062, Gibco). The MM.1S cell line was cultured in RPMI/10% FBS/1% Anti-Anti (15240-062, Gibco). A subcultivation ratio of 1:2 to 1:4 was used.

All the cell lines were kept at 37 °C in a 5% incubator with humidity and were regularly re-tested for authenticity and for mycoplasma with polymerase chain reaction (PCR) [16].

Murine Pancreatic ductal adenocarcinoma cell lines mPDAC06, mPDAC09 and mPDAC95 were isolated from tumors of Kras^G12D^ mice, which were kindly provided by Günter Schneider and Dieter Saur (Technische Universität München, Germany) [17] and cultured in DMEM/10% FBS/1% Anti-Anti (15240-062, Gibco). In- house-generated HoxB8-FL cell lines #1_HoxB8FL: *wildtype*, #2_HoxB8FL: *Iμ-HA-Bcl6* and #3_HoxB8FL: *Cxcr4^WHIM^* were isolated from bone marrow cells from the femurs of (age 10–12 weeks) [15], suspended in BBMM, pelleted by centrifugation at 450 RCF for 5 min, resuspended in 5 mL ACK lysing buffer at room temperature for 5 min, pelleted again and resuspended in BBMM. A total of 4 × 10^6^ bone marrow cells were pre-stimulated for 24 h in 2.5 mL BBMM, supplemented with rm-IL-3 (2 ng/µL), rm-SCF (10 ng/µL), IL-6 (10 ng/µL) in a 12-well plate. After removing 2 mL supernatant, cells were transduced with 1 mL ER-HoxB8 retrovirus-containing supernatant by spinoculation (1000 RCF, 90 min, 32 °C) in medium containing 8 μg/mL polybrene and incubated at 37 °C. After 24 h, all the cells were centrifuged at 300 RCF, washed with PBS and resuspended in 2.5 mL progenitor outgrowth medium for the generation of HoxB8–FL cells. In the following 3 weeks, progenitor outgrowth medium was changed every 2–3 days and serially passaged to new wells and even flasks after stable expansion. The cell density was always kept between 1 × 10^5^ and 1 × 10^6^ cells/mL medium. For the selection of immortalized progenitors, cells were cultured in 1 µg/mL G418 for 2 weeks. Cells were collected for two continuous passages with a 10-passage interval (P 5, P 15 of wild-type, *Iμ-HA-Bcl6* and *Cxcr4^WHIM^* HoxB8-FL cell line) for DNA extraction. The cells were counted, collected as pellets and re-suspended slowly and carefully at the concentration of 5 × 10^6^ cells/mL in freezing medium: 90% FCS + 10% DMSO. Defreezing/recultivation: In order to reduce the toxic effect of cryo-protectants produced during the thawing process, (liquid nitrogen) preserved cryo-vials were incubated in 37 °C water bath and quickly transferred into a 15 mL tube with pre-warmed medium. The cells were centrifuged, resuspended with 5 mL fresh medium and immediately transferred into a T25 flask and cultivated. After culturing for 3–6 days, 5 × 10^6^ cells were collected for subsequent analysis (DNA/RNA isolation).

### 2.6. Differentiation of Immortalized HoxB8-FL Cells

To differentiate ER-HoxB8 progenitors to dendritic cells (DC) and macrophages and generate the growth curve of undifferentiated and differentiated cells, 0.2 × 10^6^ HoxB8-FL cells were washed twice (centrifugation, 300× *g*, 5 min) and resuspended in 2.5 mL RP-10 medium, RP-10 medium containing 5% Flt3L-SNT (Flt3L-supernatant), RP-10 medium containing 5% Flt3L-SNT and 1μM Estradiol, RP-10 medium containing 20 ng/mL recombinant murine macrophage colony-stimulating factor (rm M-CSF) and RP-10 medium containing 50 ng/mL M-CSF and culture for 10 days at 37 °C. Cell density was adjusted if required. Cells were counted at day 3, day 6 and day 10 to generate the growth curve. Cells were harvested at day 4 for evaluation of surface marker expression by flow cytometry analysis. Flt3L-derived DC, granulocyte-macrophage colony-stimulating factor (GM-CSF)-derived DC and M-CSF-derived macrophages were generated by cultivating HoxB8-FL cells in T25 flasks for one week in RP-10 medium containing 5% Flt3L, 20 ng/mL GM-CSF (DC), or 50 ng/mL M-CSF (macrophages), respectively. Adherent cells (DC or macrophages) were detached by trypsin treatment and used for subsequent experiments.

For the in vitro differentiation of B cells, OP9 cells were collected in 50 mL tubes for irradiation at 15Gy in Mibi (Clinac 2100 CD, Varian, Palo Alto, CA, USA) and then seeded at a petri-dish to >80% confluence in the OP9 medium. Next day, 1 × 10^6^ HoxB8–FL cells were co-cultured on irradiated OP9 cells that were seeded on a petri-dish and maintained in B cell differentiation medium. Every 2–3 days, cells were passaged onto fresh OP9 cells for 2 weeks. B-cell differentiation has been determined by fluorescence-activated cell scanning (FACS) analysis using antibodies against CD19 (# 48-0193-82, eBioscience, Santa Clara, CA, USA) and B220 (# 25-0452-82, eBioscience, San Diego, CA, USA).

### 2.7. DNA and RNA Isolation and Transcriptome Analysis

Bone marrow cells for DNA isolation were collected before transduction, and after immortalization. For RNA isolation, bone marrow cells before transduction, HoxB8-FL cell lines and their differentiated lineages (M-CSF-derived cells, GM-CSF-derived cells) were used. Briefly, DNA was isolated using the DNeasy Blood & Tissue Kits (#69506, QIAGEN, Hilden, Germany) and stored in −20 °C for DNA profiling studies. RNA was isolated using the RNeasy Mini Kit (#74106, QIAGEN, Germany) and the QIAshredder (#79654, QIAGEN, Germany) following the manufacturer’s instructions and stored at −80 °C. The RNA Integrity Numbers (RINs) of the RNA samples are accessed by using Agilent RNA 6000 Nano Kit (# 5067-1513, Agilent, Santa Clara, CA, USA) and calculated by the Agilent 2100 Expert software (version B.02.10.SI764). RNA samples with RIN > 8 were used for RNA-sequencing. For transcriptomic profiling, RNAseq was performed. Briefly, library preparation and subsequent paired-end sequencing was performed by Novogene using standard Illumina protocols. Sequencing was performed on a HiSeq2500 with a sequencing depth >20 M reads/sample. Fastq files were subsequently mapped with STAR and normalized according to [18]. Principal component analysis and cluster analysis using spearman correlation have been performed over the transcriptomic data and codes in R have been made publicly available in https://github.com/enio23/Hong_etal_2020.

### 2.8. Short Tandem Repeat (STR)-Based Multiplex PCR

A total of 9 murine STR loci, including *18-3*, *4-2*, *6-7*, *9-2*, *15-3*, *6-4*, *12-1*, *5-5*, and *X-1*, from the NCBI mouse genome build 38.1 using Basic Local Alignment Search Tool (BLAST), were selected as described [11] and are depicted in Table S1. The human STR marker *D8S1106* was also incorporated for human cell line contamination detection [11]. According to the predicted PCR product sizes, 9 murine and 1 human STR markers were distributed to four sets (Table S2). All the STR-PCR experiment was performed for 2 continuous passages of each genotype HoxB8-FL cell lines, and cells before and after freezing. PCR amplification was performed on a Mastercycler nexus X2 (Eppendorf, Hamburg, Germany), which was set up in a final volume of 20 μL reaction, containing 1 ng of mouse DNA, 2 μM MgCl2 (Applied Biosystems), 250 μM dNTPs (USB Corporation, Cleveland, OH), 1× GeneAmp PCR Gold buffer (Applied Biosystems), 0.16 mg/mL BSA (Sigma-Aldrich, Taufkirchen, Germany), forward and reverse primers (Table S1) and 1 U AmpliTaq Gold DNA Polymerase (Applied Biosystems). PCR conditions for the multiplex assay were as follows: denaturation for 11 min at 95 °C, amplification for 45 cycles of 45 s at 94 °C, 2 min at 59 °C, and 1 min at 72 °C, extension for 60 min at 60 °C, and a final soak at 25 °C [11]. PCR products were analyzed on 3% agarose gel and stained by Midori Green Advance DNA Stain (MG04, Nippon Genetics Europe GmbH, Düren, Germany).

### 2.9. Fragment Length Analysis

Amplified STR-PCR fragments were analyzed on the Agilent 2100 Bioanalyzer by using the Agilent DNA 1000 Kit according to manufacturer’s instructions [19]. Briefly, 9 μL of the gel–dye mixture was first pipetted in the specific position of the DNA chip, after pressurization of the chip, 5 μL of the internal DNA marker (Agilent) was loaded into each sample well and the ladder well, then 1 μL of the PCR product was added to each of the sample well and 1 μL of the Agilent DNA 1000 ladder, used as a sizing standard, was added into the ladder well. After electrophoresis, the amplified alleles from each sample were analyzed with the 2100 Expert software (version B.02.10.SI764). The length of each STR fragment was estimated based on the ladder and internal standards; the STR markers were identified according to their predicted size ranges (Table S2).

## 3. Results

### 3.1. Differentiation and Characterization of Conditionally Immortalized HoxB8-FL Hematopoietic Progenitors

**Figure 1:**
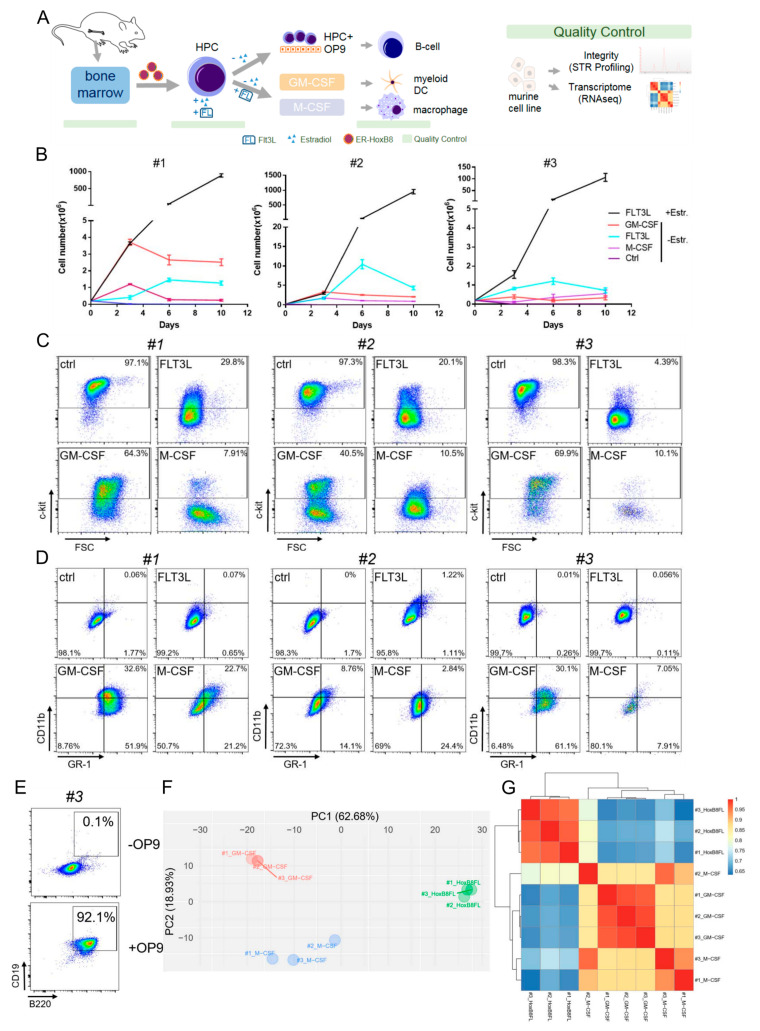
Phenotype and characterization of HoxB8-Flt3 ligand (FL)-derived myeloid and lymphoid cells. (**A**) Scheme of hematopoietic progenitor cell (HPC) generation using retroviral ER-HoxB8, and differentiation into lymphoid and myeloid cells with indicated quality controls. The scheme was drawn using motifolio drawing toolkits (Copyright © motifolio.com; 3 September 2020). (**B**) Growth curve of HoxB8-FL cells (#1: wild-type, #2 *Iμ-HA-Bcl6*, #3 *Cxcr4^WHIM^*) with the specified differentiation factors, Estr: estradiol. Error bars represent the standard deviation of three HoxB8–FL cell populations. (**C**) Fluorescence-activated cell scanning (FACS) analysis of undifferentiated HoxB8-FL cells using c-kit expression when cultivated with the specified factors. (**D**) FACS analysis of CD11b and GR1 to identify dendritic cells and granulocytes. (**E**) Co-cultivation with OP9 feeder cells induces differentiation into CD19+/B220+ B cells. (**F**) Principal component analysis of transcriptome profiles of indicated cell lines. (**G**) Cluster analysis of indicated cell lines, based on their transcriptional profile. All biological replicates were performed as technical triplicates.

For the generation of immortalized HPCs, we followed the scheme as depicted in Figure 1A as previously described [3]. Through the retroviral transduction of the fusion of the *estrogen receptor hormone binding domain* (*ERHBD*) with the coding sequence of *HoxB8* (*ERHBD-HoxB8)* and simultaneous application of both estradiol and the Flt3 ligand (FL), we were able to successfully immortalize various HPCs and derived primary HoxB8-FL HPC lines (Figure 1B). In addition to *wild-type C57Bl6/J* mice (#1) HPC, we selected two additional mouse lines and derived HoxB8-FL HPC lines from one mouse each. Both lines are associated with the development of hematopoietic disorders: *Iµ-HA-Bcl6 (#2)*, a strain that develops lymphomas with features of human diffuse large B cell lymphomas (DLBCLs) [13], and *Cxcr4^WHIM^* (#3) which shows defects in lympho-hematopoiesis [14]. The generated HoxB8-FL cells were expanded in culture for several weeks without obvious changes in growth characteristics or phenotype (Figure 1B). This model can therefore be used to generate HoxB8-driven, growth factor-dependent primary murine cell lines with HPC characteristics.

In order to characterize HoxB8-FL cells during hematopoietic cell differentiation, we deprived the cells of estradiol, which resulted in inactivation of HoxB8 and severely reduced cell growth. After withdrawal of estradiol, HoxB8-FL cells continued to grow until day 6 and did not further expand until day 10 (Figure 1B). After four days of estrogen deprivation, phenotypic changes occurred with a marked downregulation of the cytokine receptor c-Kit (Figure 1C), a receptor expressed on the surface of undifferentiated HoxB8-FL cells. We also observed a slightly increased expression of CD11b (Figure 1D). After another three days of estradiol withdrawal, the cells showed the typical phenotype of FL-derived dendritic cells (DC) [3,4]. To test whether HoxB8-FL cells have myeloid potential beyond the dendritic line, we replaced FL after the removal of estradiol by GM-CSF or M-CSF, which supports the formation of DC and granulocytes or macrophages in vitro. After a short period of reduced cell growth and limited cell death during estradiol withdrawal (Figure 1B), both cytokines supported survival, expansion and finally the formation of viable populations of differentiated cells (Figure 1D). On day 4, GM-CSF-derived HoxB8 FL cells showed the classical phenotype of GM-CSF-derived BM cells, which was characterized by a mixed population of DC (CD11b) and granulocytes (GR1) (Figure 1D). In particular, M-CSF-stimulated HoxB8-FL cells showed the characteristic adherent morphology of macrophages with a typical surface expression of CD11b and a lower expression of GR1 than GM-CSF-stimulated HoxB8-FL cells (Figure 1D).

Taken together, these data show that HoxB8-FL cells carry a differentiation potential for all major myeloid cell linages.

Lymphocyte development can be modelled in vitro by using stromal cells such as the OP9 cell line and FL support [20]. To investigate the lymphocyte potential of our HoxB8-FL cells, we co-cultivated HoxB8-FL cells (#3) with OP9 cells. We observed the differentiation into CD19^+^ B220^+^ cells, indicating the development of B cells (Figure 1E). In addition to B cells, T cells can be generated from the immortalized HoxB8-FL cells, which requires the additional expression of the Notch ligand delta-like 1 in OP9 cells [21].

To further investigate whether HoxB8-FL cells transcriptionally cluster differently after hematopoietic differentiation, we created transcriptome profiles of the three genotypes (#1, #2, #3) after differentiation with M-CSF or GM-CSF. A principal component analysis (Figure 1F) and a cluster analysis (Figure 1G) revealed significant differences between the immortalized undifferentiated immature HoxB8-FL and the differentiated counterparts.

### 3.2. Authentication of HoxB8-FL Cell Lines by STR Profiling

**Figure 2:**
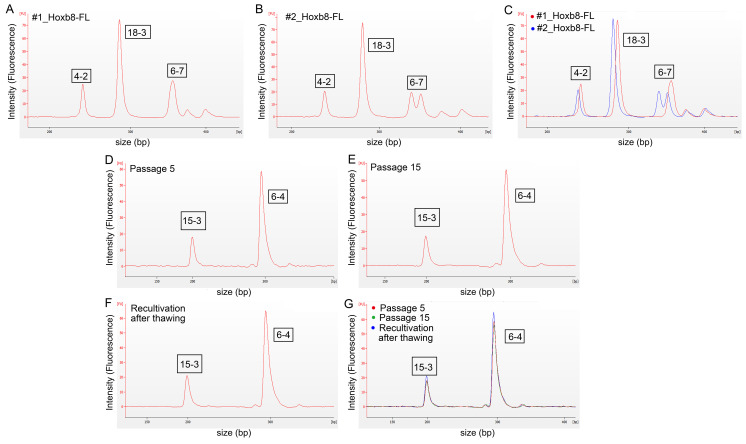
Electropherograms for the analysis of fragment lengths, detection of individual short-tandem repeat (STR) profiles from murine HoxB8-FL cell lines. (**A**) STR amplicons of the cell line #1_HoxB8FL using primer set 1. (**B**) STR amplicons of the cell line #2_HoxB8FL using primer set 1. (**C**) Overlay of (**A**,**B**). (**D**) Proof of reproducibility of STR profiling after passage 5 of HoxB8FL cells using primer set 3 (STR locus 15-3; 6-4). (**E**) Proof of reproducibility of STR profiling after passage 15 of HoxB8FL cells. (**E**) Proof of reproducibility of STR profiling after recultivation of cryo-conserved HoxB8FL cells. (**G**) Overlay of plots displayed in (**D**–**F**).

Stringent quality controls are critical to reproducible and replicable research. To assess the integrity of the generated cell lines in terms of origin and cross-contamination, we established a microfluidics-based analysis (Agilent Bioanalyzer 2100) for STR profiling as stringent quality control. For the authentication and generation of unique STR profiles for the clear discrimination of individual mouse samples, we performed experiments with the corresponding primer pairs listed in Table S1 based on tetranucleotide repeats that are stable even at high passage numbers. These PCR-generated amplicons (Table S2) allow for the differentiation between individual mice of the same subspecies [11]. Currently, the most commonly used technology for the authentication of human cell lines is capillary electrophoresis, which is used to perform fragment length analysis from STR-PCR [22]. We transferred this technology to a microfluidic-based lab-on-chip system (Bioanalyzer 2100, Agilent) [23]. For this purpose, we designed different multiplex PCRs with the primer combinations as shown in Table S2. For two HoxB8-FL cell lines (#1_HoxB8FL and #2_HoxB8FL) electropherograms are shown in Figure 2A–C; the corresponding STR profiles of all three lines are listed in Table 2.

We compared freshly isolated bone marrow with HoxB8-FL cell lines over a cultivation period of 15 passages and could confirm stable STR profiles even after the cultivation of late passages (Figure 2D–G, Table 2 and Table S3).

**Figure 3:**
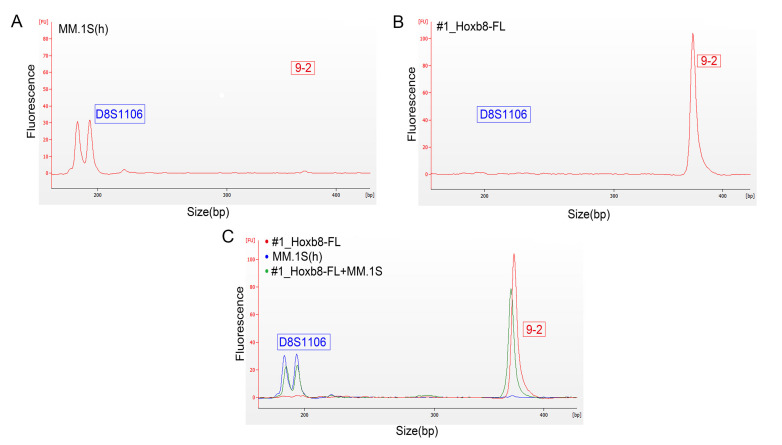
Electropherograms for the analysis of fragment lengths and the detection of cross-contamination human MM.1S cells and murine HoxB8-FL cell lines. (**A**) STR amplicon of the human STR marker D8S1106 in human MM.1S cells (primer set 2). (**B**) STR amplicon of the murine STR marker 9-2 of #1_HoxB8FL cell line using primer set 2. (**C**) The murine #1_HoxB8-FL MM.1S cell line including 1:1 ratio of DNA from human MM.1S cell line (red: human marker, green: murine marker). FU = Fluorescence Units; bp: base pairs; s: seconds. All biological replicates were performed as technical triplicates.

To investigate cross-contaminations with human cell lines, we incorporated one human STR marker that amplifies outside the murine marker in primer set 2 (Table S2). To test this for mouse-human cross-contamination, we mixed the human cell line MM.1S with the murine #1_HoxB8FL cell line with a final concentration of 1 ng of total DNA in a 1:1 ratio. We tested the murine and human cell lines separately and detected only respective murine or human peaks (Figure 3A–C). The mixture of both species revealed both peaks in one sample (Figure 3C).

Thus, using STR profiling in a microfluidic-based lab-on-chip system allows for the authentication of primary mouse cell lines with regard to single mouse origin, and for the detection of cross-contamination between species.

### 3.3. Validation of Microfluidics-Based STR Profiling in Primary Murine Pancreatic Cancer Cell Lines

Since the STR analyses were successfully performed for authentication within a stringent quality control program in hematopoietic HoxB8-FL cell lines, we next analyzed further murine cell lines for validation purposes. In this validation cohort, we profiled three murine pancreatic ductal adenocarcinoma (PDAC) cell lines generated from primary Kras-driven tumors [24]. All cell lines bear a single *Kras^G12D^* mutation as a driver mutation. Again, we could show that STR profiling reliably generated individual and cell-line-specific STR profiles (Table 3). None of the analyzed cell lines showed matches in their STR profiles in the microfluidics-based lab-on-chip analysis method. Using the Software 2100 Expert, we were able to perform FLA of the generated murine cell lines derived from the same genetic background. This enabled us to differentiate between individual donor mice and to rule out cross-contamination with human cells.

## 4. Discussion

The gold standard to authenticate human cell lines is STR-based DNA profiling (ASN-0002), and numerous journals and granting agencies require authentication of cell lines prior to the publication and funding [25]. In this article, we describe a microfluidics-based electrophoresis method for STR-based DNA profiling of “in house”-generated murine hematopoietic progenitor cell lines, which was then validated in primary murine solid cancer cell lines. In addition to conventional genotyping, this low-cost method can be used to ensure authenticity of murine cell lines, which adds to produce reproducibility and replicability of experimentations.

STR fragments were analyzed using the DNA 1000 chip on the Agilent Bioanalyzer 2100. Here, a fluorescence dye intercalates in double stranded DNA, capable of detecting a sizing resolution down to 5 bp for fragments ranging between 25 and 1000 bp [19]. The product size range of primers used is between 130 and 515 bp [11], indicating that amplicons can be separated in this setting. Independent studies have shown that this method can be used as an alternative to standard capillary electrophoresis for the STR-based DNA profiling of human cells [23,26]. We therefore established multiplexing PCRs of nine STR markers to detect up to three markers in one well for primary murine cell lines. Direct comparison of the samples confirmed distinct profiles of all six murine cell lines, indicating that these nine markers are sufficient to differentiate between primary murine cell lines derived from single mice. Implementation of an additional human marker in our multiplex assay further enables identification of human cross-contamination.

## 5. Conclusions

The misidentification and cross-contamination of cell lines that might affect reproducibility and replicability remain a serious issue in biomedical research. Genetic profiling for the traceability of samples is highly recommended for the quality assessment of biobanks [27,28] but is cost and time intensive. Especially for complex cell culture systems, their functionality should be sufficiently checked. For this purpose, we performed differentiation experiments by flow cytometry and RNAseq to verify the identity and the properties of the HPC cell lines. This first validation step is essential to generate a functional living biobank. In a second step, cell line authentication methods are necessary to ensure the integrity of the generated lines. We are convinced that the presented low-cost authentication method can increase the reproducibility of scientific results and improve the tracking of “in house”-generated cell lines through routine monitoring for cross-contaminations.

## Figures and Tables

**Table 1:** Media composition.

Medium	Concentration	Media/Additive	Catalog No.	Storage
RP-10 Medium		RPMI1640	Gibco: 21875-034	4 °C
	10%	FBS (not heat inactivated)	Gibco: 10270-106	−20 °C
	0.10%	Mercapto Ethanol	Gibco: 31350-010 50 mM	4 °C
	1%	Antibiotic Antimycotic	Gibco: 15240-062	−20 °C
Progenitor Outgrowth Medium		RPMI1640	Gibco: 21875-034	4 °C
	10%	FBS (not heat inactivated)	Gibco: 10270-106	−20 °C
	0.10%	Mercapto Ethanol	Gibco: 31350-010 50 mM	4 °C
	1%	Antibiotic Antimycotic	Gibco: 15240-062	−20 °C
	1 µM	β-estradiol	Sigma: E-2758 (stock 100 mM)	−20 °C
	5%	FLT3L-SNT	from B16 melanoma cell line	−20 °C
B-Cell Differentiation Medium		RPMI1640	Gibco: 21875-034	4 °C
	10%	FBS (not heat inactivated)	Gibco: 10270-106	−20 °C
	0.10%	Mercapto Ethanol	Gibco: 31350-010 50 mM	4 °C
	1%	Antibiotic Antimycotic	Gibco: 15240-062	−20 °C
	1 µM	β-estradiol	Sigma: E-2758 (stock 100 mM)	−20 °C
	5 ng/mL	FLT3L	R & D Systems: 427-FL	−20 °C
	25 ng/mL	SCF	R & D Systems: 455-MC	−20 °C
	7 ng/mL	IL-7	R & D Systems: 407-ML	−20 °C
BBMM (Basal Bone Marrow Medium)		IMDM	Gibco: 12440-053	4 °C
	30%	FBS (not heat inactivated)	Gibco: 10270-106	−20 °C
	0.20%	Mercapto Ethanol	Gibco: 31350-010 50 mM	4 °C
	0.50%	Antibiotic Antimycotic	Gibco: 15240-062	−20 °C
	1%	Glutamine	Corning: 25-005-CV	−20 °C
	0.50%	BSA	Sigma-Aldrich: B6917	4 °C
HF2 Buffer		ddH2O	Ampuwa: 250166	RT
	30%	FBS (not heat inactivated)	Gibco: 10270-106	−20 °C
	0.50%	Antibiotic Antimycotic	Gibco: 15240-062	−20 °C
	1%	HEPES	Gibco: 15630-049	4 °C

**Table 2:** STR profiles of 3 HoxB8-FL with indicated repeats.

Cell Line	18-3	4-2	6-7	9-2	15-3	6-4	12-1	5-5	X-1
#1_HoxB8FL (wild-type)	16	19	19	17	22	19	15	16	23
#2_HoxB8FL (*Iμ-HA-Bcl6*)	15	18	15, 18	15	20	18	16	17	24
#3_HoxB8FL (*Cxcr4^WHIM^*)	16	19	19	15	22	19	16	17	24

**Table 3:** STR profiles of 3 pancreatic ductal adenocarcinoma (PDAC) cell lines with indicated repeats.

Cell Line	18-3	4-2	6-7	9-2	15-3	6-4	12-1	5-5	X-1
mPDAC06	17, 18	18	20	16	20, 22	17	16, 20	14, 16	20, 24
mPDAC09	17, 19	17, 19	20, 23	18, 22	20	18	16	16, 17	18, 25
mPDAC95	17	19, 19	17	18, 20	21	19	16	16, 17	26

## Supplementary Materials

The following are available online at https://www.mdpi.com/2227-9059/8/12/590/s1, Table S1: STR loci of the reference genome GRCm38.p6 from C57BL/6J (Mus musculus). Table S2: Primer sets used for multiplexing polymerase chain reactions (PCRs). Table S3: Consistent STR profiles of HoxB8-FL cells from 2 continuous passages and after the freeze–thawing cycle.
