# Supplementary material for: Authentication of Primary Murine Cell Lines by a Microfluidics-Based Lab-On-Chip System
Source: Biomedicines. 2020 Dec 9;8(12):590. doi: 10.3390/biomedicines8120590 (PMC7763653; doi:10.3390/biomedicines8120590)
Supplement: Supplementary file 1 [file biomedicines-08-00590-s001.pdf]

Supplementary Materials

Table S1. STR loci of the reference genome GRCm38.p6 from C57BL/6J (Mus musculus).

STR	Repeat Motif	Primer	Product Size [bp]	Repeats	Primer Concentration (μM)
18-3	$ATCT_{[n]}$	A: TCTTTCTCCTTTTGTGTCATGC	290	[17] = 17	0.200
		B: GTCAAAGTTGGGGTTACAGAATG			
4-2	$GATA_{[n]}GATA_{[n]}$	A: AAGCTTCTCTGGCCATTTGA	238	[13][5] = 18	0.100
		B: GTTCATAAACTTCAAGCAATGACA			
6-7	$CTAT_{[n]}$	A: AGTCCACCCAGTGCATTCTC	348	[17] = 17	0.075
		B: GCATGTGGCTGGTATGCTGTT			
9-2	$GATA_{[n]}GATA_{[n]}$	A: GGCTCTCTCACACCTCATCC	357	[14][3] = 17	0.080
		B: GTCCATGAATCCAGACATTCC			
15-3	$GATA_{[n]}GATA_{[n]}$	A: TCTGGGCGTGTCTGTCATAA	197	[12][9] = 21	0.060
		B: GTTCTCAGGGAGGAGTGTGCT			
6-4	$GATA_{[n]}GATA_{[n]}GATA_{[n]}$	A: TTTGCAACAGCTCAGTTTCC	295	[2][14][3] = 19	0.100
		B: GAATCGCTGGCAGATCTTAGG			
12-1	$GATA_{[n]}GATA_{[n]}$	A: CAAAATTGTCATTGAACACATGTAA	225	[13][3] = 16	0.200
		B: GCAATGGTCAAGAAATACTGAAGTACAA			
5-5	$TATC_{[n]}$	A: CGTTTTACCTGGCTGACACA	282	[17] = 17	0.300
		B: GATGCTTGCCTGTTCCTAGC			
X-1	$GATA_{[n]}GATA_{[n]}$	A: GGATGGATGGATGGATGAAA	403	[13][12] = 25	0.300
		B: GAAGGTATATATCAAGATGGCATTATCA			

Table S2. Primer sets used for multiplexing polymerase chain reactions (PCRs).

Multiplex PCR	STR Locus	Predicted Product Size [bp]
Set 1	18-3	281–313
	4-2	217–248
	6-7	333–515
Set 2	9-2	318–360
	D8S1106	136–147
Set 3	15-3	157–222
	6-4	276–311
	D4S2408	336–360
Set 4	12-1	222–259
	5-5	258–298
	X-1	357–442

Table S3. Consistent STR profiles of HoxB8-FL cells from 2 continuous passages and after the freeze–thawing cycle.

STR Locus	*Iμ-HA-Bcl6*			*CXCR4^{WHIM}*		
	Passage 5	Passage 15	After thawing	Passage 5	Passage 15	After Thawing
18-3	286	285	288	286	283	285
4-2	241	240	242	241	239	240
6-7	347, 357	344, 355	348, 359	356	351	353
9-2	369	370	370, 380	368	368	368
15-3	197	200	200	202	199	199
6-4	292	295	294	295	295	294
12-1	223	223	224	225	224	224
5-5	280	281	283	282	281	280
X-1	394, 419	394, 421	396, 418	397, 414	396, 418	396, 416
